# A review of cell-type specific circuit mechanisms underlying epilepsy

**DOI:** 10.1186/s42494-024-00159-2

**Published:** 2024-06-01

**Authors:** Peilin Zhao, Xiaomi Ding, Lini Li, Guohui Jiang

**Affiliations:** 1 Institute of Neurological Diseases, Affiliated Hospital of Clinical School of Medicine, North Sichuan Medical College, Nanchong, Sichuan 637000 China; 2 Nanomedicine Innovation Research and Development Transformation Institute, Affiliated Hospital of Clinical School of Medicine, North Sichuan Medical College, Nanchong, Sichuan 637000 China; 3 Department of Neurology, Affiliated Hospital of Clinical School of Medicine, North Sichuan Medical College, Nanchong, Sichuan 637000 China

**Keywords:** Epilepsy, Circuit mechanisms, Cell-type specific, Neuromodulatory

## Abstract

Epilepsy is a prevalent neurological disorder, yet its underlying mechanisms remain incompletely understood. Accumulated studies have indicated that epilepsy is characterized by abnormal neural circuits. Understanding the circuit mechanisms is crucial for comprehending the pathogenesis of epilepsy. With advances in tracing and modulating tools for neural circuits, some epileptic circuits have been uncovered. This comprehensive review focuses on the circuit mechanisms underlying epilepsy in various neuronal subtypes, elucidating their distinct roles. Epileptic seizures are primarily characterized by the hyperactivity of glutamatergic neurons and inhibition of GABAergic neurons. However, specific activated GABAergic neurons and suppressed glutamatergic neurons exacerbate epilepsy through preferentially regulating the activity of GABAergic neurons within epileptic circuits. Distinct subtypes of GABAergic neurons contribute differently to epileptic activities, potentially due to their diverse connection patterns. Moreover, identical GABAergic neurons may assume distinct roles in different stages of epilepsy. Both GABAergic neurons and glutamatergic neurons with long-range projecting fibers innervate multiple nuclei; nevertheless, not all of these circuits contribute to epileptic activities. Epileptic circuits originating from the same nuclei may display diverse contributions to epileptic activities, and certain glutamatergic circuits from the same nuclei may even exert opposing effects on epilepsy. Neuromodulatory neurons, including cholinergic, serotonergic, dopaminergic, and noradrenergic neurons, are also implicated in epilepsy, although the underlying circuit mechanisms remain poorly understood. These studies suggest that epileptic nuclei establish intricate connections through cell-type-specific circuits and play pivotal roles in epilepsy. However, there are still limitations in knowledge and methods, and further understanding of epileptic circuits is crucial, particularly in the context of refractory epilepsy.

## Introduction

Epilepsy is a prevalent neurological disorder, with epidemiological surveys indicating that over 70 million individuals worldwide are impacted by this condition [1]. Approximately 30% of individuals with epilepsy suffer from refractory or drug-resistant epilepsy [2, 3]. Epilepsy is characterized by recurrent and unpredictable seizures that can cause varying degrees of damage to the nervous system [4]. Due to the intimate association between epileptic nuclei and emotional, cognitive, and other functions, comorbid mental disorders often coexist in patients with epilepsy, leading to impairments in their cognitive, learning, and social abilities [5].

The regulation of neuronal activity is a dynamic process in which the interaction between different neurons maintains the excitation-inhibition balance of the central nervous system. The mechanism of epilepsy remains incompletely understood, but a hallmark feature is the over-synchronized discharge in neural networks [6]. It is widely acknowledged that the disruption of excitation-inhibition balance in epileptic nuclei during seizures primarily manifests as a loss of inhibitory control over glutamatergic neurons, resulting in excessive excitation and synchronous discharge within the focal area. Consequently, this aberrant activity can propagate throughout the brain, leading to widespread synchrony and perturbation of normal brain function. Epileptogenesis involves multiple nuclei located throughout the cortex [7], basal ganglia [8], hippocampus [9], thalamus [10, 11], midbrain [12, 13], and cerebellum [14, 15]. The precise connectivity among neurons in the nervous system is crucial for maintaining normal physiological activity. Numerous studies indicate that epileptic seizures arise from disruptions in these connections [16]. One hypothesis posits that epilepsy propagates through the brain's neural network and interacts with distal nodal circuits. Modulating these nodes may potentially modify epilepsy transmission [17]. Epileptic patients have a network of connections in the brain associated with seizures, and by selectively interrupting specific pathways (such as the thalamus or striatum to the frontal lobe), may enable patients to achieve long-term freedom from seizures [18]. One of the principles of epilepsy surgery or deep brain stimulation (DBS) for epilepsy is to desynchronize the epileptogenic neural network by influencing the activity of key nodes in the epilepsy network [19]. Specific neurons form close interconnections with other neurons through rich input and output circuits, and the excitation-inhibition balance of these circuits profoundly impacts the development of epilepsy. Different epileptic nuclei display diversity in composition and connection, constituting different modes of information transmission. Some nuclei have relatively homogeneous types of neurons, such as the anterior group of the dorsal thalamus (ANT), which is a key nucleus for DBS in the treatment of epilepsy [20, 21]. The ANT mainly contains glutamatergic neurons, and their activity increases during seizures [22]. On the other hand, nuclei such as the reticular nucleus of the thalamus (RT) are primarily composed of GABAergic neurons [10]. Most epileptic nuclei are heterogeneous, including the cortex and hippocampus, which consist of different types of GABAergic neurons and glutaminergic neurons. Information is transmitted mainly through long-range projecting glutamatergic neurons, while interaction between glutamatergic and GABAergic neurons in local areas is also crucial for epileptic activity. In addition to GABAergic neurons and glutaminergic neurons, neuromodulator neurons such as cholinergic, serotonergic (5-hydroxytryptamine, 5-HT), dopaminergic (DA), and noradrenergic (NE) neurons also play important roles in epileptic activity [23].

Rich connections are formed among various types of neurons in different nuclei, and specific circuits within these connections play crucial roles in epileptic activity. Understanding the mechanism of these circuits is essential for comprehending the onset and progression of epilepsy. In recent years, advancements in neural tracing and modulating tools, particularly the application of optogenetic and chemogenetic methods [24–26], have enabled us to investigate the mechanisms of epileptic circuits in vivo, leading to continuous elucidation of cell type-specific epileptic circuits. This review paper examines current research on epilepsy circuitry from an excitation-inhibition balance perspective, focusing on the functional circuits formed by different types of neurons involved in epilepsy. Furthermore, it proposes prospects for research on epileptic circuits.

## Tools in epileptic circuits research

### Optogenetics

Optogenetics is a technique that combines optical and genetic techniques to finely regulate cell activity in the brain in vivo [27]. Researchers utilize molecular biology techniques or viral vectors to introduce exogenetic photosensitive protein genes into living cells. This facilitates the expression of photosensitive channel proteins on the cell membrane. By irradiating the cells with specific wavelengths of light, researchers can control the activation and shutdown of photosensitive channel proteins. This modulation of channel activity leads to changes of cell membrane potential, resulting in depolarization or hyperpolarization [28]. The first described channelrhodopsins (ChRs), including ChR1 [29] and ChR2 [30], were identified in *Chlamydomonas reinhardtii*. When these ChRs are expressed in the targeted cell and illuminated with bule light (470 nm), they pump cation such as Na^+^ from the extracellular medium into the cell and mediate depolarization and activation of the target cell [30]. Similarly, halorhodopsin from the archaea *Natronomonas pharaonis* (NpHR) can be expressed on the target cell. When the target cell was illuminated with yellow light (580 nm), chloride ions from the extracellular medium were transported into the cell and mediate hyperpolarization with consecutive silencing of the target cell [31]. Optogenetic technology has been widely used in neuroscience to realize the real-time regulation of neurons in free-moving animals and explore the function of specific circuits. In recent years, optogenetic technology has also been widely used in epilepsy research, clarifying the circuit mechanisms in various brain regions such as hippocampus, thalamus, and basal ganglia (reviewed in [24, 32, 33]). Traditional optogenetic regulation relies on an optical fiber to connect the experimental animal and laser, which can impede its motor ability and pose challenges in certain behavioral tests. To address this limitation, Yang et al. had developed a wireless optogenetic system based on electromagnetic induction principles in 2021 [34]. This system incorporates a charging coil and control chip into a fingertip-sized optogenetic stimulator that is directly embedded in the head of experimental animals, enabling stimulation of different light wavelengths as required. These advancements eliminate the constraints imposed by optical fiber and allow simultaneous regulation of multiple animal interactions during experimental behavior studies, thereby expanding the application potential of optogenetic technology in neuroscience.

### Designer receptors exclusively activated by designer drugs (DREADDs)

DREADDs are chemogenetic platforms based on G protein-coupled receptors (GPCRs) [35]. By modifying different GPCRs to deliver synthetic proteins, the modified receptors can only be selectively activated or inhibited by specific synthetic compounds. This activation or inhibition then triggers the corresponding GPCRs signaling pathway, resulting in different excitatory changes in the cell. DREADDs, which are activated by clozapine-N-oxide (CNO), can selectively act on different GPCR cascades. Under normal physiological conditions, the human muscarinic acetylcholine receptor subtype M3 (hM3) can bind to the endogenous neurotransmitter acetylcholine (Ach), and then couple with GQ-type GPCRs to participate in the GQ-type signaling pathway. Human muscarinic acetylcholine receptor subtype M4 (hM4) can bind to acetylcholine (Ach) and then couple with GPCRs of Gi to play a role in Gi signaling pathways. The two conserved sites on the modified hM3 and hM4 are mutated, rendering them unable to bind to acetylcholine, but can bind to exogenous CNO efficiently. The two mutated receptors are named hM3Dq and hM4Di. Under the action of CNO, hM3Dq can activate neuronal activity, while hM4Di exhibits an inhibition effect [36]. DREADDs are one of the indispensable tools in the study of neural circuit mechanisms and have been widely used in studies related to epileptic circuits (reviewed in [25]). Compared with optogenetics, DREADDs do not need the pre-embedding of ceramic cores and impose relatively minimal trauma to experimental animals. DREADDs have a long and sustained regulation of neuronal activity and are more suitable for behavioral experiments that require long-term observation, such as the study of chronic epilepsy [22].

### GPCR activation-based neurotransmitter/neuromodulator sensors

Neurotransmitters and neuromodulators are important messengers in the synaptic transmission of the nervous system, and their disorder contributes to various diseases including Alzheimer's disease [37], Parkinson's disease [38], and epilepsy [23]. Calcium imaging is used to detect signal activity in postsynaptic neurons [39], but the spatio-temporal variations of different neurotransmitters and neuromodulators in specific activities remain unclear. By leveraging GPCRs binding neurotransmitters as a scaffold for the sensors, Li’s team has successfully developed a new series of genetically encoded fluorescent sensors of neurotransmitters and neuromodulators since 2018 [40, 41]. These sensors involve the fusion and modification of specific human neurotransmitter receptors with fluorescent proteins at the molecular level. The underlying principle involves embedding the fluorescent protein with a specific neurotransmitter receptor, and the binding of the receptor to the neurotransmitter will cause the conformational change of the receptor to be converted into a fluorescent signal [40]. Through virus injection, transfection and other techniques, the genetically encoded probe can be expressed in cells or mouse brains, and the real-time change of neurotransmitter concentration can be observed by imaging technology [42]. These neurotransmitter and neuromodulator sensors have extremely high sensitivity, molecular specificity, precise spatial resolution and sub-second response speed [43], which is superexcellent for us to evaluate the activity change of different neurotransmitter and neuromodulator in epilepsy in vivo.

## The circuit mechanism of GABAergic neurons in epilepsy

Studies have demonstrated significant alterations in the transcriptome of major subsets of glutamatergic and GABAergic neurons among patients with epilepsy [44]. Calcium signal records in the cortex reveal [7] distinct temporal recruitment patterns of different neuronal types during epileptic seizures. Initially, various types of GABAergic neurons, including parvalbumin (PV), somatostatin (SST), and vasoactive intestinal peptide (VIP) neurons, are employed in epileptic seizures, followed by glutamatergic neurons.

### The local epileptic circuits of GABAergic neurons

**Fig. 1:**
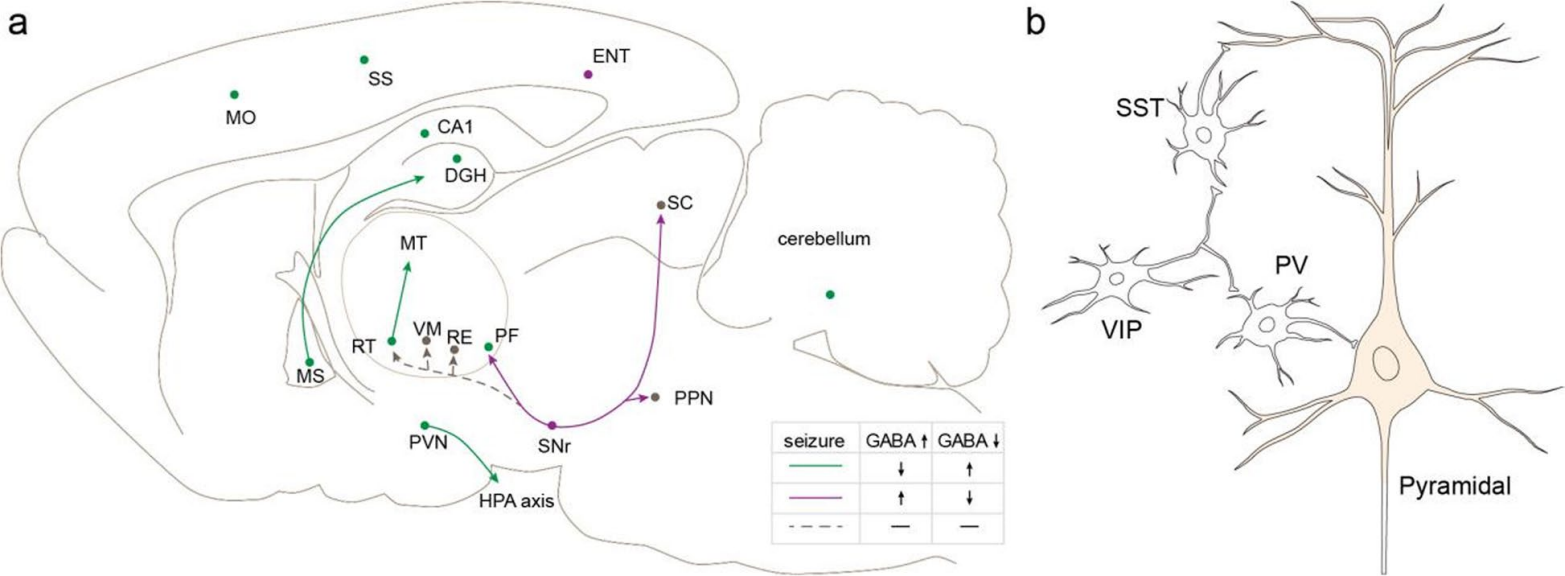
GABAergic circuits in epilepsy. **a** The uncovered GABAergic epileptic circuits in the whole brain. The green arrows and dots represent GABAergic circuits, whose activation inhibits epileptic activity or exacerbates epileptic seizures upon inhibition, while the magenta arrows and dots exhibit the opposite effect. The dotted lines represent circuits with no significant effect on epileptic activity. **b** The connection patterns among different GABAergic neurons and pyramidal neurons in the cortex

Most GABAergic neurons in the cortex and hippocampus have local projections, regulating the activity of neighboring neurons. Activation of these neurons within epileptic circuits often exhibits antiepileptic effects. Optogenetic activation of PV neurons in the barrel cortex may exert an antiepileptic effect by inhibiting the activity of pyramidal neurons [45] (Fig. 1a). Activation of dentate gyrus/hilus (DGH) GABAergic interneurons can significantly suppress the spread of ictal seizures and effectively rescue behavioral deficits in kainate-exposed animals [46]. Chemogenetic activation of distinct subpopulations of inhibitory neurons in the hippocampus decrease epileptic activity [8, 47–49]. Activation of PV neurons (including Purkinje cells) in the middle region of the cerebellum reduce the secondary frequency of epilepsy [50]. Similarly, the activity of GABAergic neurons in the parafascicular nucleus (PF) bidirectionally regulates seizures [13].

Altered connections of GABAergic neurons in epileptic nuclei contribute to the excitation-inhibition imbalance in the epileptic brain. Impaired GABAergic transmission is considered one of the potential mechanisms underlying refractory epilepsy [51]. On one hand, damage to presynaptic GABA receptors on neurons within focal areas reduces their ability to receive GABAergic information, thereby inducing an imbalance between excitation and inhibition. Some drugs used in clinical epilepsy treatment primarily enhance the function of GABA receptors (such as benzodiazepines [52]). On the other hand, the loss of GABAergic neurons in focal nuclei due to pathological changes is also crucial for the excitation-inhibition imbalance [53]. The reduction of local GABAergic neurons decreases the inhibition of local circuits, which compromises the precise regulation of excitation-inhibition balance. For instance, selective silencing of PV neurons in the hippocampus can trigger the onset of spontaneous epilepsy [54]. In this regard, inducing differentiation through stem cell transplantation or glial cells [55] into GABAergic neurons can alleviate epilepsy. Moreover, chemogenetic inhibition of CA1 PV/SST neurons enhances epileptic activity [8].

GABAergic neurons exhibit diverse subtypes and activity patterns in epilepsy. In the cortex [7], optogenetic inhibition of VIP neurons increases the seizure threshold and reduces seizure duration. Conversely, inhibition of PV and SST neurons consistently reduces seizure duration albeit with different effects on seizure activity. PV neurons in the subiculum are more prone to depolarization in secondary epilepsy [49]. The antiepileptic effect originating from activated cholinergic circuits projecting from the medial septal nucleus (MS) to the hippocampus could be reversed by inhibiting hippocampal SST neurons rather than PV neurons [8]. These functional differences may be attributed to distinct connectivity mechanisms of different types of GABAergic neurons [9] (Fig. 1b). Studies conducted on the cortex have demonstrated that PV neurons primarily exert inhibitory control over pyramidal neurons, playing a crucial role in regulating cortical output. Meanwhile, PV neurons are regulated by VIP neurons, SST neurons, and other PV neurons [56]. SST neurons predominantly inhibit the distal dendrites of pyramidal neurons while also preventing PV neurons from receiving inhibition from VIP neurons. However, direct connections between different SST neurons are infrequent, and their inhibitory effect is weaker compared to that of PV neurons, which may potentially contribute to cortical plasticity [57]. VIP neurons primarily inhibit SST and PV neurons in the cortex to relieve the inhibition of excitatory projection neurons in the cortex [58], with minimal direct connections with pyramidal neurons.

Additionally, the extensive interactions among different types of neurons suggest that not all activated epileptic GABAergic circuits suppress epilepsy [59]. The activation of various subtypes of GABAergic neurons in the entorhinal area (ENT) aggravates the severity of hippocampal epilepsy [60–62]. In cortical dysplasia-related seizures, optogenetic activation the SST neurons in the dentate gyrus facilitates seizure generalization while PV neurons retain an inhibitory role [63]. Furthermore, certain GABAergic neurons may exhibit diverse functions during different stages of epilepsy. Optogenetic activation of GABAergic neurons in the subiculum suppresses the activity of pyramidal neurons and thus delays secondary epileptogenesis [49]. However, once mice develop stable secondary seizures, optogenetic activation of GABAergic neurons in the subiculum paradoxically enhances the depolarization of GABAergic signals and exacerbates secondary epileptic seizures.

### The long-range projection epileptic circuits of GABAergic neurons

In addition to local regulation, some GABAergic neurons project long-range axons to other brain regions and are involved in epilepsy (Fig. 1a). Within the MS, GABAergic neurons extensively regulate the neuronal activity of different hippocampus regions. Hristova et al. have demonstrated that activation of MS GABAergic neurons reduces the duration of epilepsy through wireless optogenetics [64], while another study found no contribution of these GABAergic circuits to epilepsy [8]. The RT serves as the primary GABAergic nucleus in the thalamus with abundant axonal projections throughout different thalamic nuclei. Optogenetic activation of the GABAergic circuits projecting from the RT can inhibit epileptic-related behaviors [11], including weakening limbic system epilepsy by inhibiting midline thalamus (MT) neuron activity [10]. Interestingly, the RT neurons innervate bilateral MT and optogenetic activation of the contralateral but not ipsilateral RT projections to the MT attenuate amygdala-kindled seizures [10]. Corticotropin-releasing hormone (CRH) containing neurons are a subtype of GABAergic neurons [65] that play important roles in depression-like behaviors via the hypothalamic-pituitary-adrenal (HPA) axis. In chronically epileptic mice, activation of CRH neurons in the paraventricular nucleus (PVN) increases seizure susceptibility while inhibition is sufficient to decrease seizure susceptibility and depression-like behaviors [66]. In rats with audiogenic epilepsy, the lesion of the superior colliculus (SC) can disrupt epileptic activity resulting from the abnormal activity of small and medium-sized GABAergic neurons [12]. Auditory seizures originate in the central nucleus of the inferior colliculus, propagate to the external nucleus of the inferior colliculus, and project to the deep layer of the SC (DLSC) [67], subsequently spreading to the pontine reticular nucleus.

GABAergic neurons in the substantia nigra reticular nucleus (SNr) also have long-range axons and are involved in epileptic activities. Unlike MS or RT, activation of SNr GABAergic neurons and their GABAergic fibers in the PF exacerbates epileptic seizures [13]. Notably, the PF is a heterogeneous nucleus containing both GABAergic neurons and glutaminergic neurons that bidirectionally regulate epileptic activity. Therefore, within epileptic circuits projecting from the SNr to PF, activation of SNr PV neurons preferentially inhibits PF GABA neurons to release their inhibition to glutamatergic neurons and promote epileptic seizures. Apart from the PF, the SNr PV neurons also innervate other thalamic nuclei including the RT, ventral medial nucleus of the thalamus (VM), and nucleus of reunions (RE) without contribution to epileptic activity. In addition to ascending projection, SNr GABAergic neurons also project descending to the mesosphere and DLSC and pedunculopontine nucleus (PPN). Notably, inhibition of SNr GABAergic cells and their axons in the DLSC can inhibit pentatetrazole-induced general epilepsy, forebrain-induced focal epilepsy, gammabutylactone-induced absence epilepsy, and audiogenic epilepsy in genetically epileptic susceptible rats, while inhibition of axonal terminals in the PPN only reduces absence epilepsy but exacerbates pentatetrazole-induced general epilepsy [68].

Generally, distinct subtypes of GABAergic neurons participate in epileptic activity primarily through the action of different neurons within local circuits, while certain long-range projecting GABAergic neurons regulate other nuclei via diverse projecting circuits. Furthermore, although most activated GABAergic neurons exhibit an antiepileptic role, some activated GABAergic neurons promote the development of epilepsy, such as the SNr PV neurons.

## The circuit mechanism of glutamatergic neurons in epilepsy

The hyperexcitability of glutamatergic neurons is a direct contributor to epileptic seizures and inhibiting these neurons in the epileptic nuclei can reduce seizure activity [22, 69, 70]. Glutamatergic neurons with long-range axons are responsible for transmitting information from cortical and hippocampal regions to other brain regions.

### The diverse roles of different glutamatergic collaterals in epilepsy

**Fig. 2:**
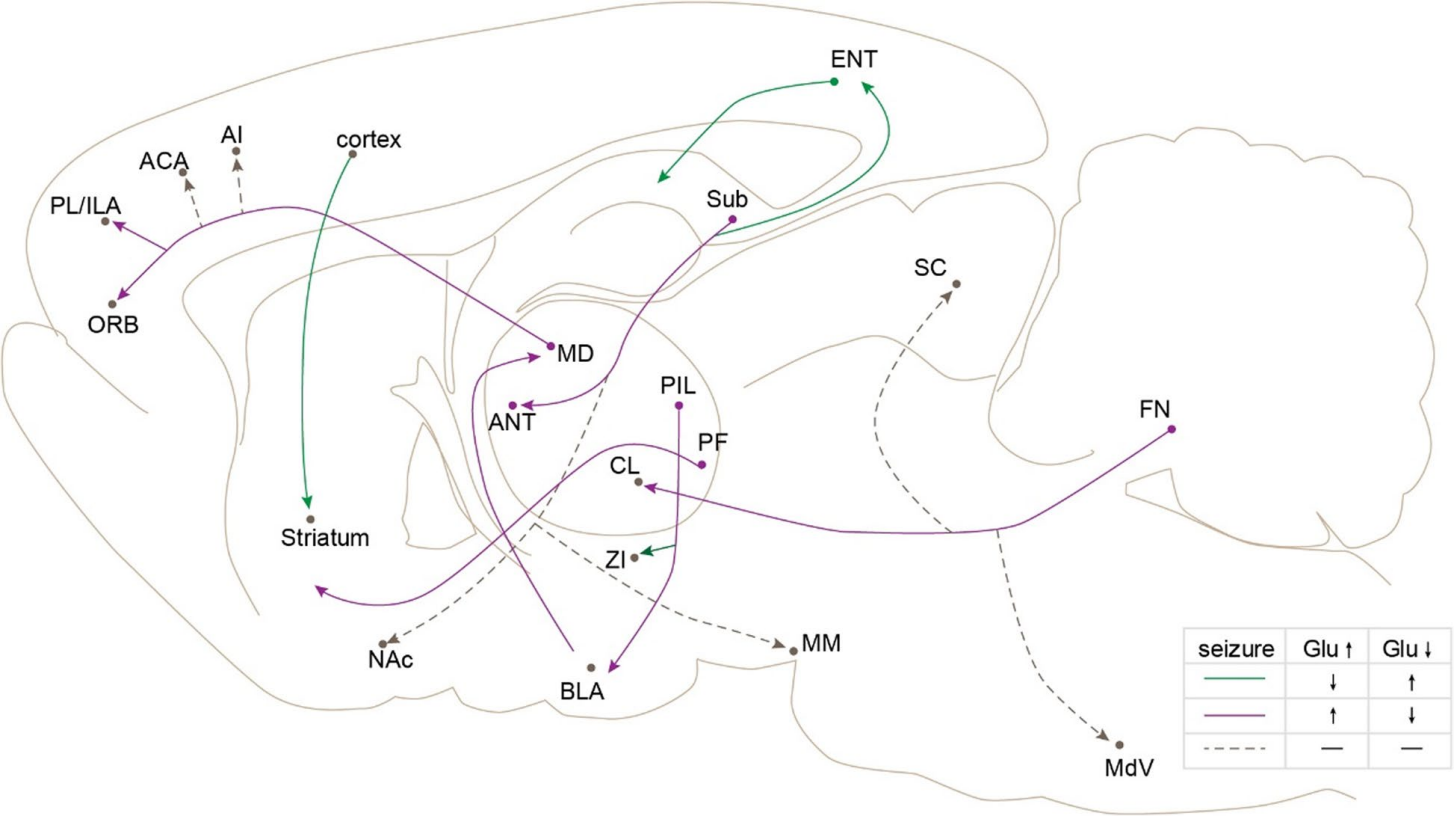
The uncovered glutamatergic epileptic circuits in the whole brain. The magenta arrows and dots represent glutamatergic circuits, whose activation exacerbates epileptic seizures or inhibits epileptic activity upon inhibition, while the green arrows and dots exhibit the opposite effect. The dotted lines represent circuits with no significant effect on epileptic activity

Excitatory projecting neurons always exhibit extensive axon branching, enabling simultaneous modulation of multiple brain regions. Investigations conducted in the hippocampus [22], thalamus [71], cerebellum [14], and other areas have demonstrated that diverse projecting circuits within epileptic nuclei may exert distinct roles in epilepsy pathogenesis. On the one hand, not all glutamatergic circuits originating from the epileptic nucleus contribute to epileptogenesis (Fig. 2). For instance, activation of circuits projecting from the cerebellar parietal nucleus (FN) to the centrolateral thalamic nucleus (CL) exerts inhibitory effects on seizures, whereas regulation of circuits projecting to the SC or medullary reticular region (MdV) does not impact epileptic activity [14]. In the amygdala-kindled model [72], chemogenetic or optogenetic inhibition of glutamatergic projections from the basolateral amygdala nucleus (BLA) to the medial thalamic nucleus (MD) eliminated epileptic behavior and epileptiform electrical emission. Similarly, chemogenetic inhibition of MD projections to the anterior limbic cortex (medial prefrontal cortex) effectively abolished epileptic seizures. However, inhibitory effects on other circuits projecting to the frontal cortex were only partially observed in the orbitofrontal cortex and inferior limbic cortex, while no significant impact was observed in the anterior cingulate area (ACA) and agranular insular area (AI). Clinical studies have also demonstrated that non-pathological circuits in an epileptic brain do not exhibit recurrent abnormal activity [71].

On the other hand, distinct projecting circuits originating from the same nucleus may exhibit opposing effects on epilepsy (Fig. 2). For instance. Fei et al. [22] have discovered that the subiculum-ANT circuits regulate seizures in a bidirectional manner; activation of these circuits promotes seizures while inhibition alleviates them. As the primary efferent nuclei of the hippocampus, subiculum neurons widely projected to the ENT, nucleus accumbens (NAc), and medial mammillary nucleus (MM) besides ANT [9]. Activation of circuits projecting from the subiculum to the ENT inhibited epileptic seizures whereas those targeting NAc and MM did not participate in epileptic activity [22]. Similarly, glutamatergic neurons expressing calentomental protein in the posterior intralaminar thalamic nucleus (PIL) are activated during epileptic seizures. Inhibition of their circuits projecting to the lateral amygdala decelerates epilepsy progression, while inhibition of their circuits projecting to the zone incerta (ZI) promotes epileptic seizures [73].

One possible explanation for the above phenomenon is that distinct circuits originate from different subgroups of neurons within the same nuclei. Additionally, retrograde tracing results have demonstrated a clear separation in the distribution of neurons among these circuits [14, 22, 73], indicating specific groups of neurons are involved in epileptic activities within epileptic nuclei. This hypothesis has also been supported by findings in the SC, where optogenetic activation of deep/intermediate layers of SC neurons inhibits epileptic behaviour and electrical activity in models of pentatetrazole and area tempestas (forebrain/complex partial seizures) [74]. Conversely, modulation of other areas within the SC does not impact epileptic activity. However, the segregation between projecting circuits is not absolute as neurons of specific circuits also have collaterals in other brain regions. Even if the co-projecting regions are also involved in epileptic activity, the regulation of their collaterals may not directly impact epileptic activity [14]. This suggests that the diverse roles of different projecting circuits in epilepsy are not only derived from distinct subgroups but also from their relationship with specific downstream targets.

### The anti-epileptic effects of activated glutamatergic circuits

Similar to GABAergic neurons, the correlation between glutamatergic circuit activities and epileptic activities is not always positive (Fig. 2). For instance, inhibiting the glutamatergic circuits from the PIL to ZI promotes the seizure of epilepsy [73]. Activation of glutamatergic circuits in the ENT has shown inhibitory efforts on epilepsy [22]. Furthermore, activation of glutamatergic neurons in the ENT reduces the severity of hippocampal seizures [75] primarily by enhancing the activity of hippocampal GABAergic neurons to suppress hippocampal glutamatergic neuron function [60]. Similarly, injury of glutamatergic circuits original from the cortical neurons to the striatum in mice with deletion of *Stxbp1* or *Scn2a* genes leads to the absence epilepsy primarily may result from loss of glutamatergic transmission from the cortical neurons to striatal fast-spiking interneurons. Chemical inhibition of these circuits in wild-type mice also induces the absence seizures and spasms. Additionally, optogenetic activation of glutamatergic neurons in the cerebellar fastigial nucleus inhibits seizures [76].

## The circuit mechanism of neuromodulatory neurons in epilepsy

**Fig. 3:**
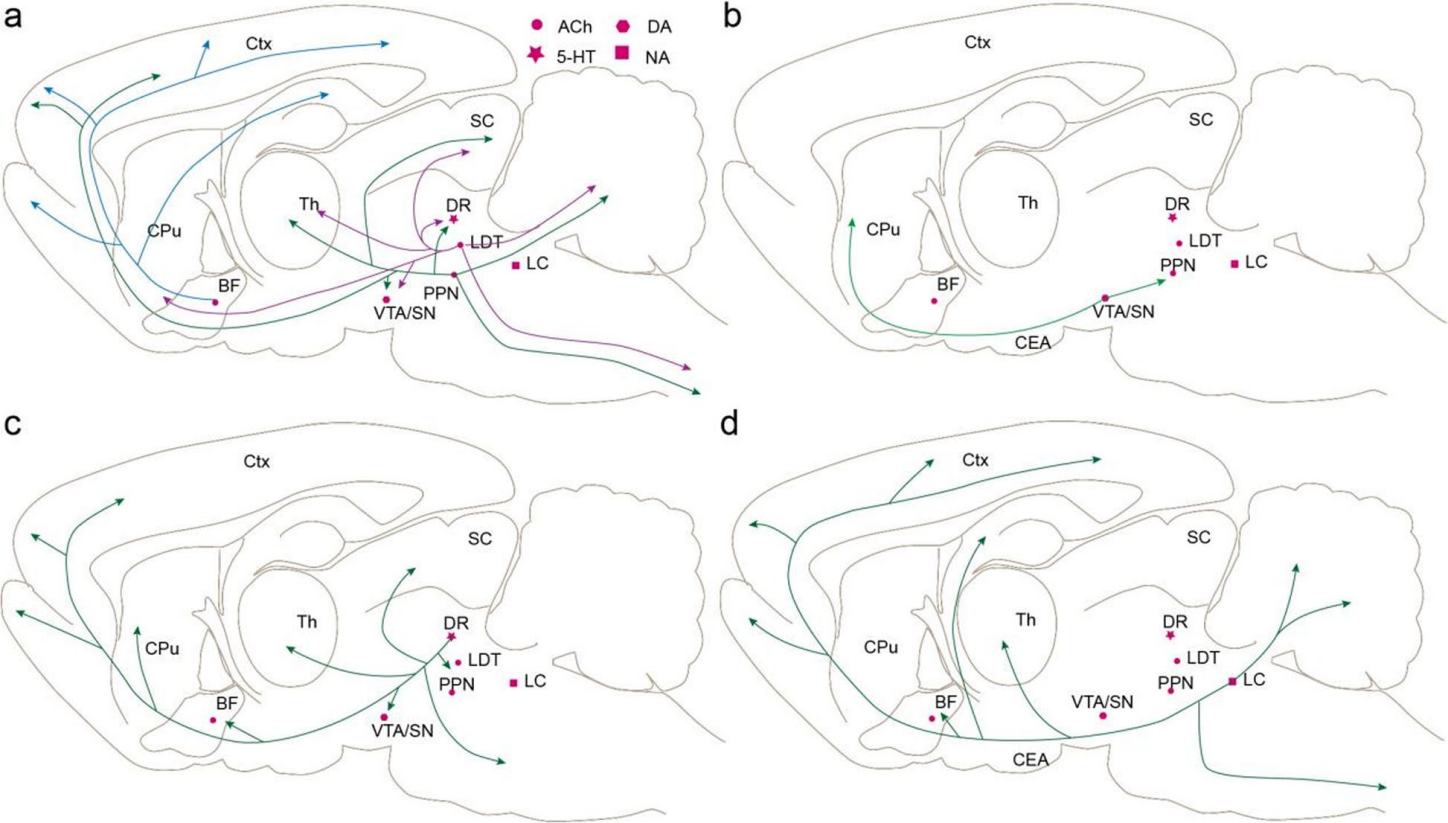
The whole-brain projection of different neuromodulatory neurons. **a** The whole-brain projection of cholinergic neurons in the BF, PPN, and LDT. **b** The whole-brain projection of DA neurons in the VTA. **c** The whole-brain projection of 5-HT neurons in the DR. **d** The whole-brain projection of NE neurons in the LC. Abbreviation: *Ctx* cortex; *CPu* caudate putamen; *BF* basal forebrain; *Th* thalamus; *SC* superior colliculus; *DR* dorsal raphe nucleus; *LDT* laterodorsal tegmental nucleus; *PPN* pedunculopontine nucleus; *LC* locus ceruleus; *VTA* ventral tegmental area; *SN* substantia nigra; *CEA* central amygdala

In addition to GABAergic neurons and glutaminergic neurons, neuromodulatory neurons such as cholinergic, serotonergic, noradrenergic, and dopaminergic neurons, also play crucial roles in epileptic activity [23]. These neuromodulatory neurons are concentrated in a few nuclei with abundant connections with other brain regions and contribute to various functions of the central nervous system (Fig. 3).

Cumulative evidence suggests that cholinergic signaling plays a crucial role in the modulation of epilepsy [77]. The basal forebrain (BF) contains abundant cholinergic neurons and serves as the main source of acetylcholine in the cortex and hippocampus [78]. In temporal lobe epilepsy mice, activation of cholinergic circuits projecting from the MS to the hippocampus stops electrical spikes during epileptic seizures by enhancing the activity of inhibitory neurons within the hippocampus [8]. Importantly, this antiepileptic effect could be specifically reversed through chemogenetic inhibition of hippocampal SST neurons rather than PV neurons. Besides the hippocampus, the BF cholinergic neurons project abundant axons to the cortical areas, amygdala, and other epileptic nuclei [79] with some cholinergic circuits implicated in epilepsy [77]. Cholinergic neurons are also concentrated in some brainstem nuclei such as the PPN and laterodorsal tegmental nucleus (LDT). During epilepsy, reduced firing was found in selected PPN cholinergic neurons [80, 81]. In focal seizures, optogenetic activation of PPN cholinergic neurons leads to an increase in cortical γ oscillations and a decrease in δ activity [82], indicating that reduced subcortical cholinergic arousal may contribute to cortical dysfunction during epileptic seizures. PPN cholinergic neurons primarily regulate cortical activities via an indirect pathway projecting to the thalamus [83] with some cholinergic collaterals directly to the cortex [84, 85]. Enlarged perinuclear bodies were observed in both PPN and LDT cholinergic neurons along with increased cholinergic projections to the thalamus in KA-kindled models [86].

The DA system plays a crucial role in the pathogenesis of Parkinson's disease [87]. Emerging evidence suggests that the DA neurons also contribute to epileptic activity [88]. Cavarec’s [89] study found that increased dopamine transporter and D3 receptor expression and activity already occured before the onset of seizure in the absence epilepsy-prone rats. The midbrain DA neurons projected most axons ascending to the striatum and some axons descending to the midbrain [90], including the PPN, which is involved in epileptic activities [80, 81, 91].

Serotonin is an important neuromodulator in the central nervous system. 5-HT neurons in the brainstem have abundant axons in the whole brain [92] and play a crucial role in controlling breathing, cardiac function, and arousal—all of which are impaired during seizures [93]. In the pentatetrazole-induced epilepsy models, optogenetic activation of 5-HT neurons in the dorsal nucleus raphe (DR) effectively inhibited tetanic seizures in most mice while significantly reducing the probability of seizure induced respiratory arrest, without altering the latency and duration of clonic seizures [94]. Conversely, increased activity of DR 5-HT neurons was observed in hippocampal kindled mice; optogenetic inhibition rather than activation of these neurons significantly delayed epilepsy formation—particularly at later stages [95].

The NE neurons in the locus ceruleus (LC) were impaired in some epileptic patients. Vagus nerve stimulation has been used as an adjunctive treatment for drug-resistant epilepsy due to its ability to activate the LC-noradrenergic system [96]. LC NE neurons have rich projections in ascending and descending pathways [97]. In the ascending pathways, a lesion of the LC converts sporadic seizures evoked by microinfusion of bicuculline into the anterior piriform cortex of rats into limbic status epilepticus [98]. In the descending pathways, recent studies suggest that the noradrenergic pathway from the LC to the heart is implicated in modulating sudden unexpected death in epilepsy [99].

In general, neuromodulatory neurons play a pivotal role in the manifestation of epileptic activity. These neuromodulatory neurons exhibit concentrated localization within specific nuclei while projecting extensively throughout the entire brain, establishing intricate connections with epileptogenic nuclei. Nevertheless, the precise circuit mechanisms underlying their involvement in epilepsy remain elusive.

## Conclusion and outlook

The orderly connectivity of neural networks is fundamental for maintaining normal physiological activities. Increasing evidence suggests that epilepsy is a neurological disease characterized by abnormal circuits and the formation and development of epileptic circuits serve as an important basis for the onset of epilepsy. Overactivation of glutamatergic neurons and decreased activity of GABAergic neurons are the main manifestations underlying epilepsy, recent advances in cell type-specific circuitry have provided deeper insights into the mechanisms underlying epileptic circuits.

Firstly, the epileptic circuit exhibits cell-type specificity. Generally, seizures are characterized by the overactivation of glutamatergic circuits and inhibition of inhibitory circuits. Activation of inhibitory neurons and inhibition of glutamatergic neurons in epileptic circuits show anti-epileptic effects. Most epileptic circuits align with this characteristic; however, some exhibit the opposite performance. For instance, the activation of SNr PV neurons promotes seizures, and the activation of ENT glutamatergic neurons inhibits them. This unusual performance may be related to the information integration pattern within their cell type-specific connections in the epileptic circuits. For example, SNr PV neurons exert direct control over the activity of GABAergic and glutaminergic neurons in the PF, with inhibition of GABAergic neurons promoting epileptic seizures. However, the circuitry involving glutamatergic neurons did not exhibit a significant impact on epileptic activity. This suggests that GABAergic neurons in the PF hold greater priority within the SNr-PF circuit during epileptogenesis. Similarly, the activation of ENT glutamatergic neurons suppresses epileptic activity by stimulating hippocampal GABAergic neuron function. Furthermore, distinct subtypes of GABAergic neurons may play varying roles in epilepsy based on their connectivity.

Secondly, epileptic circuits exhibit nucleus-specificity and not all connections between epileptic nuclei are implicated in epilepsy. On the one hand, projective neurons, both excitatory and inhibitory, possess abundant branches that regulate activity across multiple brain regions. However, different projection circuits of the same nucleus do not necessarily contribute to epilepsy; for instance, while SNr is involved in epilepsy through innervating PF GABAergic neurons, its projection to VM and RE thalamic nuclei does not play a role in epilepsy. Similarly, MD glutamatergic neuron projections to ORB are involved in epilepsy but projections to cortical regions such as ACA and AI are not. Notably, different circuits of the same nucleus even have opposite effects in epilepsy, for example, activation of sub glutamatergic neurons and their axons to the ANT promotes the onset of epilepsy but the circuits to ENT activation exhibit anti-epileptic effects. This may be attributed to distinct projection circuits originating from different subpopulations. However, the separation is not absolute, as single-neuron reconstructions reveal a certain degree of co-projection among various projection circuits. On the other hand, resolved epileptic circuits demonstrate that a given nucleus can be regulated by multiple epileptic nuclei, each with differing roles in epileptic activity. For instance, projection circuits from the FN to SC do not impact epileptic activity; conversely, inhibiting the SNr GABAergic circuit projecting to SC suppresses epileptic activity. Finally, neuromodulatory neurons have abundant fibers and are widely involved in epileptic activity; however, their circuit mechanism remains poorly understood. In addition to the different types of neurons, glial cells also play important roles in epileptic activity [100, 101]. Recently, Purnell et al. have reviewed [102] the role of astrocytes in epilepsy by intervening the excitation-inhibition balance of the neural circuits.

Overall, although the mechanisms underlying many epileptic circuits have been elucidated, numerous unresolved questions persist. For instance, the circuit mechanisms of pivotal epileptic nuclei such as the cortex, amygdala, ANT, and SC remain unexplained. Studies have revealed close interconnections among several epileptic nuclei like the cortex and thalamus; however, it remains unclear whether these interconnected circuits are implicated in epilepsy and what their precise mechanism of action is. Additionally, for heterogeneous nuclei, understanding the recruitment sequence and interaction of different neuron types within epileptic networks and determining if there is consistency across various nuclei are crucial aspects for comprehending epilepsy formation and seizure mechanisms.

## Data Availability

Not applicable.

## Abbreviations

5-TH: 5-hydroxytryptamine
ACA: Anterior cingulate area
AI: Agranular insular area
ANT: Anterior group of the dorsal thalamus
BF: Basal forebrain
BLA: Basolateral amygdala nucleus
CL: Centrolateral thalamic nucleus
CNO: Clozapine-N-oxide
CRH: Corticotropin-releasing hormone
DA: Dopaminergic
DBS: Deep brain stimulation
DGH: Dentate gyrus/hilus
DLSC: Deep layer of the superior colliculus
DR: Dorsal raphe nucleus
ENT: Entorhinal area
FN: Fastigial nucleus
HPA: Hypothalamic-pituitary-adrenal
ILA: Infralimbic area
LC: Locus ceruleus
LDT: Laterodorsal tegmental nucleus
MD: Medial thalamic nucleus
MdV: Ventral medullary reticular
MM: Medial mammillary nucleus
MO: Motor areas
MS: Medial septal nucleus
MT: Midline thalamus
NAc: Nucleus accumbens
NE: Noradrenergic
ORB: Orbital area
PF: Parafascicular nucleus
PIL: Posterior intralaminar thalamic nucleus
PL: Prelimbic area
PPN: Pedunculopontine nucleus
PV: Parvalbumin
PVN: Paraventricular nucleus
RE: Nucleus of reunions
RT: Reticular nucleus of the thalamus
SC: Superior colliculus
SNr: Substantia nigra, reticular part
SS: Somatosensory areas
SST: Somatostatin
VIP: Vasoactive intestinal peptide
VM: Ventral medial nucleus of the thalamus
ZI: Zone incerta
